# Prevalence of Fibromyalgia in chronic kidney disease pre-dialysis patients: Experience from a Tertiary Care Renal unit in Pakistan

**DOI:** 10.12669/pjms.37.7.4474

**Published:** 2021

**Authors:** Zara Khan, Rubina Naqvi

**Affiliations:** 1 Zara Khan, Registrar, Department of Nephrology Sindh Institute of Urology and Transplantation (SIUT), Karachi, Pakistan; 2 Prof. Rubina Naqvi, Department of Nephrology Sindh Institute of Urology and Transplantation (SIUT), Karachi, Pakistan

**Keywords:** Fibromyalgia Syndrome (FMS), Chronic Kidney Disease (CKD), Stages III and IV, Pre-Dialysis, Pakistan

## Abstract

**Background and Objective:**

Fibromyalgia syndrome (FMS) is a well-established medical problem which gives rise pain at various sites, fatigue, sleep disturbances, poor memory and definitely affects quality of life. Its prevalence in chronic kidney disease (CKD) is scarcely reported, thus we aimed to assess this condition and report its prevalence in our population.

**Methods:**

The current study was carried out in all adult CKD stage III and IV patients registered from January 2020 to July 2020 at outpatient department of a tertiary care renal institution in Karachi, Pakistan. This is a cross sectional study where prevalence of FMS was assessed by interviewing and examining patients according to established criteria for FMS. All data and laboratory parameters were recorded on a proforma and statistical analysis was done on SPSS version22.0.

**Results:**

During the study period of six months, 161 patients with CKD stage III and IV were registered. Among these 81 male and 80 were females. Mean age was 47.12±9.27 (range 21-60) years. There were 22 (13.66%) patients found to have FMS. Mean Widespread Pain Index (WPI) score was 5.68±4.36 (range 1-16), while severity scale (SS) 2a was 3.17±1.78 (range 1-9) and SS2b 2.04±0.96 (range 1-5) was recorded.

**Conclusion:**

From Pakistan prevalence of FMS has never been published. As this syndrome affects quality of life of patients, its recognition and proper management is immensely required.

## INTRODUCTION

Fibromyalgia syndrome (FMS) is a condition characterized by high levels of pain, sleep disturbances and fatigue combined with a general increase in memory disturbances leading to psychological distress.^1^ The FMS frequently effects the quality of life.

The chronic non-malignant pain has been reported to affect 19-24% of the general population.^2^,^3^ and quality of life of their relatives especially female relatives is impaired.^4^ The prevalence of fibromyalgia in general population estimated to be 5.4% in A meta-analysis published from UK.^5^

Fibromyalgia used to be defined by American College of Rheumatology as Widespread Pain for at least three months and presence of at least 11 of 18 tender points on examination.^6^ In 2010, a new-criteria was proposed that focused more on multiple symptoms and was further modifies to require only self-report of symptoms.^7^

Chronic Kidney Disease (CKD)is an important health problem worldwide causing heavy burden on patients and their families with an estimated overall prevalence of 8-16%.^8^ A number of common complaints are similar to FMS symptoms in CKD patients such as pain, chronic fatigue, sleep disturbances, psychiatric co-morbidities such as depression and anxiety and restless leg syndrome.^9^ Therefore, it is important to rule out other disorders from FMS and provide appropriate treatment to such patients.

There is limited literature available for FMS prevalence in pre-dialysis CKD patients and none published from Pakistan. Fibromyalgia is a difficult disorder to diagnose and this study is designed to assess the frequency of fibromyalgia in patients with chronic kidney disease (Stage-III and IV) so that by diagnosing and appropriately treating Fibromyalgia, we can improve patient quality of life and spread awareness among the physicians regarding this underdiagnosed and untreated condition.

## METHODS

This cross-sectional study was carried out at outpatient department of Sindh Institute of Urology and Transplantation, Karachi, Pakistan. Study period was six months starting from January to July 2020. Sample size was calculated from reported frequency of 18.4% in pre-dialysis CKD patients^10^, with margin of error 6% at 95% confidence level there were 154 patients were required. Inclusion criteria was all adults from both gender CKD stage-III and IV patients. While exclusion criteria were patients on dialysis or having other co-morbids which can cause fatigue for example anemia, hypothyroidism, hepatitis, vitamin D deficiency, secondary hyperparathyroidism and hyperuricemia.

CKD staging was done according to KDIGO guidelines.^11^ Symptom Widespread Pain Index (WPI) enlists 19 areas of the body where fibromyalgia patients have pain. WPI was recorded by taking history and examining these sites. WPI score can be between 0-19. Severity Scale (SS) was recorded according to American College of Rheumatology (ACR) criteria for fibromyalgia. The maximum score on SS is 12. A person was labelled to have fibromyalgia if criteria one or two are met. Whereas, as criteria one is WPI of ≥ 7 with SS of ≥ 5 and criteria two is WPI 3-6 and SS ≥ 9. SS 2a which address fatigue, waking unrefreshed and cognitive symptoms, while 2b addresses other systematic symptoms experienced by patient during previous week when they were interviewed. This is in accordance with defined criteria by ACR.

After getting approval from institutional research and ethics review committee and informed consent from patients the data was recorded. (ERC approval number SIUT-ERC-2020/A-183). All patients were interviewed in their native language and information was collected about the site of the pain during previous week and their WPI score was calculated accordingly. SS was assessed as per the symptoms mentioned in the Criteria (SS 2a and 2b). An annexure describing the scoring system is provided with this manuscript.

In all patients Complete Blood Picture (CBC), inflammatory markers like ESR and CRP, viral Serology for hepatitis B and C, Bone Chemistry (that includes Serum Calcium, Phosphorous, Alkaline Phosphatase and Albumin) and Uric Acid values was recorded.

### Statistical analysis

All the data was entered and analyzed in SPSS version 22. Mean and standard deviation was determined for continuous variables such as age and duration of symptoms. Categorical variables like gender, stages of CKD (III or IV), WPI score, SS 2a, SS 2b and fibromyalgia present or absent was determined and presented as frequencies and percentages. Effect modifier such as age and gender were stratified and association was assessed with fibromyalgia in CKD Stage III and IV by using parametric student t test and non-parametric Mann Whitney U test (as skewed distribution was seen in data) and normality test using Kolmogorov- Smirnov test. P-value of < 0.05 was considered as significant.

## RESULTS

During the study period of six months 161 patients with stage III and IV were registered in outpatient, mean age of these patients was 47.12±9.27 (range 21-60) years. There were 81 male and 80 female patients. Hepatitis B and C status and other inflammatory markers were negative in all studied population. Other demographic and laboratory parameters are given in Table-I. There were 22 (13.66%) patients found to have FMS. Mean WPI score was 5.68±4.36 (range 1-16), while SS 2a was 3.17±1.78 (range 1-9) and SS2b 2.04±0.96 (range 1-5) was recorded. Vitamin D levels are not available for all patients, therefore not described in table. Different causes of CKD in both groups are described in Table-III.

**Table I:** Demographic and laboratory parameters (n=161).

*Parameters*	*Mean ±STH*	*Median*	*Range*
Age (years)	47.12± 9.27	48	21-60
Hemoglobin (g/dl)	11.10± 1.54	11	9-15
Albumin (mg/dl)	2.95± 0.74	3	1.6-5.2
Calcium (mg/dl)	8.51± 0.87	8.3	6-11
Phosphorus (mg/dl)	3.14± 0.98	3.1	1.6-6
Alkaline phosphatase (U/L)	70.22± 36.77	63.5	31-224
Uric acid (mg/dl)	3.87± 1.42	3.7	1.5-7.4

**Table II:** Parameters statistical comparison among two groups. (n=161).

*Parameter*	*Fibromyalgia (n=22)*	*No Fibromyalgia (n=139)*	*P-value*
***Age***			
< 45 year	8	36	0.109
>45 years	14	103	
***Gender***			0.81
Male	12	68	
Female	10	71	
***CKD stages***			0.159
III	8	73	
IV	14	66	
WPI score	11.82 ± 1.94	4.44 ± 3.13	<0.001
SS score 2a	5.05 ±1.36	2.71 ± 1.45	<0.001
SS score 2b	2.45 ± 0.73	1.79 ± 0.68	<0.001

**Table III:** Causes of CKD in studied population (n=161).

*Causes*	*Fibromyalgia (n=22)*	*No Fibromyalgia (n=139)*
Renal Calculous Disease	3	25
Systemic Lupus Erythematosis	1	9
Renal Artery Stenosis	0	1
Hypertension	8	22
Glomerulonephritis	3	19
Diabetes and Hypertension	3	16
Diabetes Mellitus	0	4
Adult Polycystic Kidney Disease	1	12
Acute Cortical Necrosis	0	11
Unknown	3	20

## DISCUSSION

The diagnosis of FMS is notably clinical and is made by assessing the patients with history and clinical examination according to established criteria. In addition to diffuse pain in skeletal muscle and the physical examination finding of multiple tender points, most patients with FMS also report fatigue, muscle stiffness, pain after exertion, and sleep disturbances. The findings of the current study have reported frequency of FMS observed in 13.66% in patients with CKD stages III and IV. A study published from Turkey has reported a little higher prevalence of 18.4% in pre dialysis and 25.7% in dialysis CKD patients.^10^ A survey done in five European countries in general population has revealed the 14% prevalence of fibromyalgia.^12^ Whereas a National Health survey done in US has reported a prevalence of about 2% in general population.^13^ Another study from Turkey reveals higher prevalence in hemodialysis patients and statistically significant differences in cognitive symptoms between fibromyalgia and non-fibromyalgia groups.^14^ In yet another study published from Brazil, the prevalence was reported as 3.9% in dialysis population which was interestingly similar to general population. They observed female predominance in this study.^15^ In present study we found male to female ratio in FMS group as 1.2:1, males were slightly higher in number than female in our population.

According to an Iranian study 12.2% of 148 hemodialysis patients were suffering from fibromyalgia with higher depression and anxiety levels found in these patients.^1^ After doing search using different search engines; for any study published from our country, Pakistan; looking at fibromyalgia in chronic kidney disease patients we found no study on this particular subject, though one in patients with rheumatoid arthritis was done at a hospital in Karachi.^16^

According to the current study findings, no significant association of FMS was found with CKD stages when fibromyalgia group compared with no fibromyalgia (p value 0.159) (Table-II) The high prevalence of chronic pain in CKD population affects quality of life in adverse fashion. A published meta-analysis which includes 36 studies examining over 5,200 patients (mostly patients on dialysis) reports that over 58% of CKD patients experience chronic pain and 49% of these rates this pain as moderate or severe.^17^

In the current study, a higher frequency of FMS was noted in patients with more than 45 years of age. Somewhat similar findings were reported in a previous study in which prevalence was lowest in the 18–29 age group and higher in the people with age range of 50–59 year.^13^ Another study has reported that manifestation of fibromyalgia is predominant in 40-59 years of age.^18^ The probable reason behind higher percentage in older age group is the reduction in the physical activity and presence of comorbid conditions. It is suggested to study the health-related quality of life in these patients to assess the age associated fibromyalgia.

A comprehensive pain assessment is critical to provide an appropriate treatment plan. Identifying the underlying etiology of pain for prompt correction is both critical and ideal but does not always lead to complete pain resolution. The management of persistent pain requires a firm understanding of the underlying pathogenesis for targeted therapy rather than nonselective use of analgesics or antidepressants as well as an accurate assessment of intensity.

### Limitation of the study

We could not perform vitamin D levels in all of studied population, though we planned it at the beginning of study but social and financial reasons were hindrance in fulfilling this plan.

## CONCLUSION

Our study is first to report FMS in CKD stages III and IV from Pakistan. The frequency of fibromyalgia was found to be 13.66% in patients with CKD stage III-IV. Detection and treatment of fibromyalgia may lead to improvement in the quality of life of CKD patients. Therefore, we suggest the evaluation of the symptoms of FMS in patients with CKD.
